# Responding to the COVID‐19 pandemic: The role of occupational health services in a tertiary hospital in Singapore

**DOI:** 10.1002/1348-9585.12172

**Published:** 2020-10-15

**Authors:** Jeff Hwang, Elsie Yong, Karen Cheong, Zheng Jye Ling, Lay Hoon Goh, Fong Seng Lim, Victor Loh, Natasha Bagdasarian, Jyoti Somani, Sophia Archuleta, Judy Sng, See Ming Lim

**Affiliations:** ^1^ Occupational Health Clinic National University Hospital National University Health System Singapore Singapore; ^2^ Saw Swee Hock School of Public Health National University of Singapore Singapore Singapore; ^3^ Wellness Centre National University Hospital National University Health System Singapore Singapore; ^4^ Regional Health System Office National University Health System Singapore Singapore; ^5^ Department of Family Medicine National University Health System Singapore Singapore; ^6^ Division of Infectious Diseases Department of Medicine National University Hospital National University Health System Singapore Singapore; ^7^ Yong Loo Lin School of Medicine National University of Singapore Singapore Singapore

**Keywords:** business continuity plan, COVID‐19, hospital, infection control, occupational health

## Abstract

With coronavirus disease 2019 declared a Public Health Emergency of International Concern on 30 January 2020, occupational health services in a tertiary hospital in Singapore stepped up via a three‐pronged approach, namely, protection of individual staff, protection of staff workforce, and prevention of nosocomial spread so as to support business continuity plans. Despite the multiple new challenges brought by the COVID‐19 pandemic, the hospital's occupational health services were able to adapt and keep all employees and patients safe with strong support from senior management and close collaboration with various departments.

## 1 INTRODUCTION

Coronavirus disease 2019 (COVID‐19) was declared a Public Health Emergency of International Concern by the World Health Organization on 30 January 2020 and was subsequently characterized as a pandemic on 11 March 2020.^1^ Globally, there were over 7.9 million cases with more than 430 000 deaths at the point of writing on 16 June 2020, with the numbers increasing daily.^2^

Singapore reported its first case on 23 January 2020. Since then, the Singapore Ministry of Health (MOH) introduced a series of public health measures with additional precautionary measures introduced on 7 February 2020 in view of the emergence of local infected cases without any links to previous cases or travel history to China.^3^, ^4^ At the point of writing, a total of 40 969 cases had been confirmed in Singapore.^5^

With the large number of reported cases, there was a concern for healthcare worker (HCW) exposure and nosocomial transmission especially in light of a February 2020 report that 3387 of 77 262 COVID‐19 patients in China were healthcare workers or others who worked in medical facilities.^6^

In this paper, we illustrate the steps taken by the Occupational Health Clinic (OHC) in a tertiary hospital in Singapore during the first 3 months since 7 February 2020. OHC is an in‐house clinic set up by the hospital to manage the work‐related health and safety issues of more than 7800 staff, ensuring that they can work in a safe environment with well‐being looked after.^7^ We describe the three‐pronged approach in line with organizational and national directions, namely, protection of individual staff, protection of staff workforce, and prevention of nosocomial spread in managing staff health and safety during COVID‐19 (Table 1).

**TABLE 1:** Three‐pronged approach taken by OHC in a tertiary hospital in managing staff health and safety during COVID‐19

Approach	Actions taken
Protection of individual staff	Fitness for work assessment for new employees Vaccination against vaccine‐preventable diseases N95 mask fitting for employees in high‐risk work areas to COVID‐19 Redeployment of employees with chronic medical conditions
Protection of staff workforce	Surveillance of staff post‐travel for temperature and ARI symptoms Surveillance of staff with COVID‐19 exposure for temperature and ARI symptoms Temperature and sick leave surveillance of hospital staff
Prevention of nosocomial spread	Standardized report sick protocol for unwell staff Return‐to‐work assessment for staff who reported ARI

Abbreviations: ARI, acute respiratory illness; OHC, Occupational Health Clinic.

## 2 PROTECTION OF INDIVIDUAL STAFF

### 2.1 Fitness for work assessment for new employees

In order to support the additional public health measures implemented in the hospital such as temperature screening and closer controls of entry points into the hospital premises, temporary staff were hired at short notice. Moreover, in order to cope with the increased COVID‐19‐associated workload, new staff were recruited by various departments such as laboratory medicine and nursing. All these were in addition to the routine recruitment of new employees. All had to be reviewed by OHC for fitness for work as part of the pre‐employment requirements. To ensure safety and health of individual new staff, health status was optimized prior to deployment at various work areas, and advice on safe deployment was discussed with the reporting officer and new staff if required. Follow‐up appointments at OHC were given to new employees with relevant medical conditions to make sure that they were coping well in the first few weeks at work. In total, over the first 3 months, a total of 1389 new staff were seen by OHC (Figure ).

**FIGURE 1:**
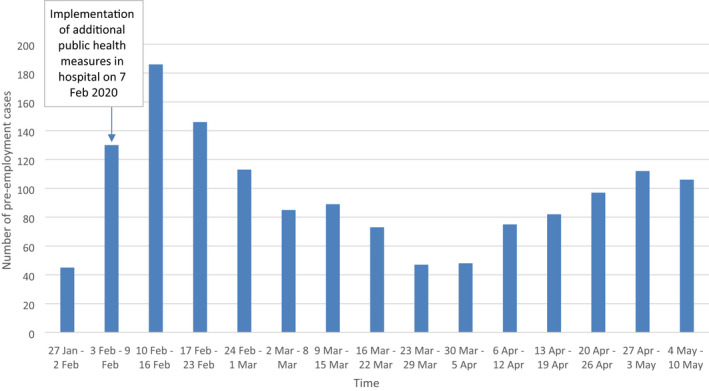
Number of pre‐employment cases seen at Occupational Health Clinic (OHC) before and after implementation of additional public health measures in hospital on 7 February 2020. There were new staff hired at short notice to cope with the demands due to new measures and increased workload. In total, over the first 3 months, a total of 1389 new staff were seen by OHC. On average, around 100 new pre‐employment cases were seen weekly since 7 February 2020, as compared to around 50 cases prior

### 2.2 Vaccination against vaccine‐preventable diseases

To safeguard the health of the new employees, OHC ensures all are adequately vaccinated against vaccine‐preventable diseases such as measles/mumps/rubella and varicella prior to starting work. For those with medical conditions that may render the healthcare workers unfit for certain vaccinations, job restrictions or re‐deployment may be required. In addition, the bi‐annual influenza vaccination exercise organized by OHC and Human Resource department each May was initiated, although without the mass vaccination events. All these were especially crucial to reduce risk of outbreaks within the hospital which might divert critical resources from COVID‐19‐related operations.

### 2.3 N95 mask fitting for employees in high risk work areas to COVID‐19 and redeployment of employees with chronic medical conditions

For employees deployed to work areas with high risk of exposure to COVID‐19 patients, N95 mask fitting had to be increased in frequency from once per week to daily, given the number of staff who needed to fitted or re‐fit as we scaled up. All staff were required to wear surgical masks in all clinical areas from 7 February 2020. OHC also helped to re‐deploy employees with chronic medical conditions at increased risk of more complicated COVID‐19 infection to non‐COVID‐19 patient areas.

## 3 PROTECTION OF STAFF WORKFORCE

Monitoring the health of the workforce within the hospital is essential to ensure that no one with acute respiratory illness (ARI) symptoms suspicious for COVID‐19 reports to work. This may potentially result in colleagues being put on leave of absence (LOA) or quarantine order,^8^ or worse still, infecting their colleagues directly. This surveillance role undertaken by OHC is all the more important given that more COVID‐19 patients were being admitted and sufficient manpower would be required to meet the demands of the increased workload.

### 3.1 Surveillance of staff post‐travel for temperature and ARI symptoms

In line with MOH suspect case criteria for COVID‐19, staff returning from high‐risk regions/countries were required to declare their travel histories and post‐travel health status to OHC and were placed on LOA or stay home notice (SHN) for the next 14 days.^8^ During this period, they had to comply with temperature and ARI symptom surveillance twice a day. At two fixed timings daily, staff would have to report their temperature and inform OHC if they developed any ARI symptoms via phone calls or short message service (SMS). If they were unwell with symptoms of ARI within the 14‐day period, they would be recalled to report to OHC for consultation and SARS‐CoV‐2 PCR testing via nasopharyngeal swab. Returning staff would only go back to work if they were completely asymptomatic during the 14‐day period.

### 3.2 Surveillance of staff with COVID‐19 exposure for temperature and ARI symptoms

**FIGURE 2:**
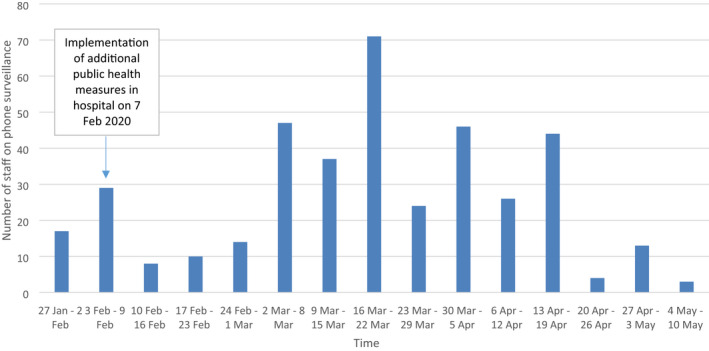
Number of staff on phone surveillance at Occupational Health Clinic (OHC) before and after implementation of additional public health measures in hospital on 7 February 2020. All staff with unprotected exposure to COVID‐19 cases in the community or in the hospital and staff returning from overseas post‐travel would be put on phone surveillance of acute respiratory illness (ARI) symptoms twice a day. If they were asymptomatic at the end of 14‐day period post‐exposure or post‐travel, they would be able to return to work without further review. However, if they reported development of ARI symptoms when under surveillance, they would be required to report sick immediately and to be swab tested for COVID‐19. Altogether, a total of 376 staff were put on symptom surveillance in the first 3 months of COVID‐19 local transmission

Employees who had unprotected exposure to COVID‐19 cases in the community or in the hospital were reviewed at OHC and risk stratified based on their exposure and duration. All exposed staff would be put under surveillance of ARI symptoms twice a day. If staff were deemed to have high‐risk exposure by MOH, they would be issued quarantine order.^8^ A decision would then be made either to issue exposed staff not on quarantine order by MOH with a LOA for 14 days or to allow staff to continue working with appropriate infection prevention measures, based on the work areas and job scopes of staff. For those who were on quarantine order/LOA and asymptomatic at the end of 14‐day period post‐exposure, they would be able to return to work without further review. However, if they reported development of ARI symptoms when under surveillance, they would be required to report sick immediately and to be swab tested for COVID‐19. This risk‐based approach in turn would prevent any potential transmission of intra‐hospital infection from the exposed staff. Altogether, a total of 376 staff were put on symptom surveillance in the first 3 months of COVID‐19 local transmission (Figure 2), of which 226 were on quarantine order/SHN/LOA.

### 3.3 Temperature and sick leave surveillance of hospital staff

OHC started working closely with the Human Resource department to ensure all staff in hospital log in temperature readings twice a day on a secured IT platform as part of the workforce health surveillance. Healthcare workers with temperature readings of greater than 37.5°C would be flagged up for further evaluation by OHC. Staff on sick leave related to ARI were closely monitored during COVID‐19 period. Technology was leveraged using an online form via a government‐developed secured IT platform (form.gov.sg) for declaration of ARI symptoms when staff reported sick, thus, allowing consolidation of daily sick leave. This allowed for prompt action by OHC to alert the department supervisor and the hospital Epidemiology team if there was any sharp increase in ARI‐related sick leave taken for further action, analysis, and trending.

## 4 PREVENTION OF NOSOCOMIAL SPREAD

### 4.1 Standardized report sick protocol for unwell staff

To reduce the risk of intra‐hospital transmission of COVID‐19 from healthcare workers, staff who were unwell with ARI symptoms were to report sick at primary care clinics and not to come to work. Teleconsultation by OHC staff was offered as a convenient alternative for staff with minor symptoms of ARI who preferred to self‐medicate.

For employees who developed ARI symptoms with COVID‐19 exposure, they were to inform OHC and to return to hospital for consult and SARS‐CoV‐2 PCR testing. The hospital made a deliberate decision for them to be seen at dedicated well‐ventilated facilities in Emergency Department rather than at OHC itself, given that the viral load and correspondingly the risk of COVID‐19 transmission may be highest at the start of the illness.^9^ A dedicated medical doctor from OHC was assigned solely to see all staff reporting sick in hospital and to perform required swab tests at Emergency Department in order to prevent the mixing of sick staff and well staff within OHC. All unwell healthcare workers would be issued with mandatory 5‐day sick leave in line with MOH’s policy.^10^ Other preventive measures included ensuring that all employees seen at OHC were wearing a mask and segregating manpower allocation for COVID‐19‐ and non‐COVID‐19–related roles.

### 4.2 Return‐to‐work assessment for staff who reported ARI

**FIGURE 3:**
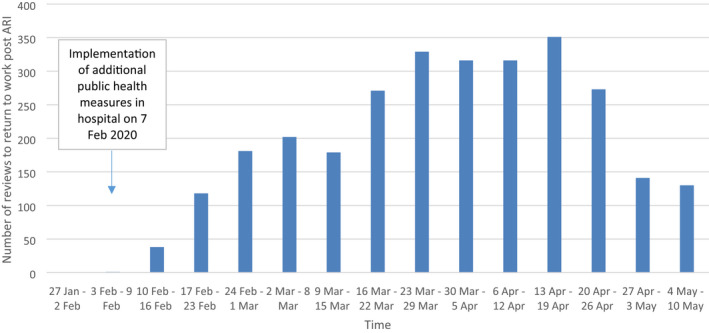
Numbers of reviews to return‐to‐work post–acute respiratory illness (ARI) before and after implementation of additional public health measures in hospital on 7 February 2020. All staff who reported sick with ARI symptoms would need to be reviewed by Occupational Health Clinic (OHC) prior returning to work. They were not on phone surveillance. A total of 2846 return‐to‐work reviews were conducted by OHC in the first 3 months since COVID‐19 local transmission in Singapore

At the end of sick leave, staff were reviewed at OHC for fitness to return‐to‐work assessment. During the review, healthcare workers who reported sick for ARI would have been swab tested for COVID‐19 if not done so prior to return to work in accordance to suspect case criteria from MOH.^11^ Any staff not recovering or not fully recovered would have sick leave extended and/or sent for re‐swabbing. A total of 2846 return‐to‐work reviews were conducted by OHC in the first 3 months since COVID‐19 local transmission in Singapore (Figure 3).

The threshold for re‐swabbing was lower for healthcare workers who were still unwell after the initial 5‐day sick leave if they had been caring for immunocompromised patients or had COVID‐19 exposure. For staff with prolonged symptoms of ARI of more than 2 weeks, they would be referred to other relevant specialists for review for safe return to work after at least two negative swabs for COVID‐19.

## 5 DISCUSSION

We have illustrated how the roles of occupational health services in a hospital have evolved and expanded during a pandemic situation. These roles remain in line with the occupational health functions under the International Labour Organisation Occupational Health Services Convention in 1985, namely, surveillance of workers’ health in relation to work, and providing advice on occupational health and protective equipment.^12^ Given that the availability of healthy healthcare workers is crucial for the business continuity of the hospital, it is important for occupational health services to maintain their capability in safeguarding the health and safety of the workforce. It is worthwhile to note that with the multiple measures put in place, we have achieved zero nosocomial transmission of COVID‐19 within the hospital till now.

### 5.1 Manpower

We faced several challenges from which useful lessons were learnt. Adequacy of manpower was one of them, since OHC had to perform extra roles in addition to the existing occupational health functions. Strong support from the hospital was critical in getting additional manpower in. It is also important to strategize continuously how things can be done more effectively and efficiently. For example, technology had been leveraged, both in the initial assessment of staff who are mildly ill requiring only rest at home, and to clear recovered staff for return to work. This allowed the OHC staff to dedicate time to other functions.

### 5.2 Communication

Ever‐changing protocols, such as who to test and who to quarantine, during the rapidly evolving situation were challenging to the operations of many clinics. We have found it useful to maintain regular communication channels with the hospital senior management and infectious disease specialists so that any new changes can be informed and implemented in a timely manner. Moreover, it is important for occupational health services to leverage our expertise to inform various key decisions. For example, at the start of the pandemic where we faced limitations with regard to the availability of swab reagents, OHC was able to work with the infectious disease specialists to come out with a risk‐based approach for swab testing. This helped us to conserve our resources in ensuring that patients who needed the test would be able to get it.

### 5.3 Health needs of staff during pandemic

Another challenge that we faced is to look after other health needs of staff in this time. While OHC is able to continue to manage healthcare workers with work‐related issues such as needle‐stick injuries and fitness to work concerns, we do recognize that staff at this time may be facing other difficulties, such as mental well‐being, maintaining healthy lifestyles such as diet and exercise, and issues related to work from home. Various departments in the hospital had been tasked to manage these issues. For example, the Human Resource department circulated a work‐from‐home guide to address some of the issues related to work from home, and the clinical departments conducted studies to assess stress and burnout among frontline staff and other hospital staff. This demonstrates that safeguarding the health and well‐being of hospital staff is never the responsibility of OHC alone, but rather the collective responsibilities of different stakeholders.

### 5.4 Technology

As we enter the “new normal” in the midst of COVID‐19 pandemic in Singapore, it is important to think how occupational health services would look like in the future. Moving forward, there will be increasing use of technology such as telemedicine facilities, and occupational health services would need to be able to leverage this. It is useful to think how technology can improve some of the existing functions such as pre‐employment screenings and post‐exposure reviews. At the same time, it is important to be mindful of the challenges and potential issues, such as completeness of a medical examination, confidentiality, and protection of medical information.

### 5.5 Work mobility

As workforce becomes more mobile, with more people working from home or working at different sites, it is worthwhile thinking about the roles of occupational health services in protecting these staff. Questions include the extent to which occupational health services should “interfere” with the workstation set‐up that staff have in their homes, whether injuries or illnesses suffered at home is considered work related, the extent which flexibility of work or increased work mobility may influence the mental well‐being of staff, and lastly, work productivity and health promotion at the new “home” workplace. While the occupational health service providers have responsibilities in managing these issues, a multi‐disciplinary team approach with other stakeholders in the hospital such as Human Resource and legal departments will be crucial to define the roles accordingly.

### 5.6 Integrated surveillance system

Lastly, although hospitals in Singapore have robust surveillance systems where staff sickness episodes and vaccination status are recorded and monitored, information is often not shared between various organizations. As demonstrated by the COVID‐19 pandemic, public health response measures are more effective when instituted earlier. Having integrated surveillance functions among the various hospitals and MOH will allow quick sharing and easy access of essential information so that any potential infectious disease outbreaks can be identified and controlled early. Occupational health services at various hospitals can have a role to play in this integrated surveillance system.

## 6 CONCLUSION

It takes concerted effort and commitment by OHC to sustain the hospital business continuity plan despite being disrupted by this unprecedented COVID‐19 pandemic. Without the strong support from senior management of the hospital and the close collaboration of various departments, it would not be possible for OHC to keep up its expanded roles efficiently while maintaining the pre‐COVID‐19 scope of work. While occupational health service aims to keep staff and patients safe via the three‐pronged approach in the hospital, it is also essential to pre‐emptively look into the challenges of the “new normal” of work brought along by COVID‐19.

## DISCLOSURE

*Approval of research protocol*: N/A. *Informed consent*: N/A. *Registry and the registration no. of the study/trial*: N/A. *Animal studies*: N/A. *Conflict of interest*: N/A.

## AUTHOR CONTRIBUTIONS

JH and SML wrote the manuscript with input and guidance from all co‐authors. JH and SML contributed equally to the writing. All authors contributed to the activities described and writing of the manuscript, and approved the final version for publication.
